# Neutrophil myeloperoxidase harbors distinct site-specific peculiarities in its glycosylation

**DOI:** 10.1074/jbc.RA119.011098

**Published:** 2019-11-12

**Authors:** Karli R. Reiding, Vojtech Franc, Minke G. Huitema, Elisabeth Brouwer, Peter Heeringa, Albert J.R. Heck

**Affiliations:** ‡ Biomolecular Mass Spectrometry and Proteomics, Bijvoet Center for Biomolecular Research and Utrecht Institute for Pharmaceutical Sciences, University of Utrecht, 3584 CH Utrecht, The Netherlands; § Netherlands Proteomics Center, 3584 CH Utrecht, The Netherlands; ¶ Department of Rheumatology and Clinical Immunology, University Medical Center Groningen, University of Groningen, 9700 AB Groningen, The Netherlands; ‖ Department of Pathology and Medical Biology, University Medical Center Groningen, University of Groningen, 9700 AB Groningen, The Netherlands

**Keywords:** myeloperoxidase, MPO, neutrophil, glycosylation, mannose-6-phosphate (M6P), mass spectrometry (MS), autoimmune disease, anti-neutrophil cytoplasmic autoantibody (ANCA), glycoproteomics, paucimannose, self-antigen, phosphomannose

## Abstract

Anti-neutrophil cytoplasmic autoantibodies (ANCAs) are directed against lysosomal components of neutrophils. ANCAs directed to proteinase 3 and myeloperoxidase (MPO) in particular are associated with distinct forms of small vessel vasculitides. MPO is an abundant neutrophil-derived heme protein that is part of the antimicrobial defense system. The protein is typically present in the azurophilic granules of neutrophils, but a large portion may also enter the extracellular space. It remains unclear why MPO is frequently the target of antibody-mediated autoimmune responses. MPO is a homodimeric glycoprotein, posttranslationally modified with complex sugars at specific sites. Glycosylation can strongly influence protein function, affecting its folding, receptor interaction, and backbone accessibility. MPO potentially can be heavily modified as it harbors 5 putative *N*-glycosylation sites (10 in the mature dimer). Although considered important for MPO structure and function, the full scope and relative abundance of the glycans attached to MPO is unknown. Here, combining bottom-up glycoproteomics and native MS approaches, we structurally characterized MPO from neutrophils of healthy human donors. We quantified the relative occupancy levels of the glycans at each of the five sites and observed complex heterogeneity and site-specific glycosylation. In particular, we detected glycosylation phenotypes uncommon for glycoproteins in the extracellular space, such as a high abundance of phosphorylated high-mannose species and severely truncated small glycans having the size of paucimannose or smaller. We hypothesize that the atypical glycosylation pattern found on MPO might contribute to its specific processing and presentation as a self-antigen by antigen-presenting cells.

## Introduction

Autoimmune diseases are characterized by the presence of autoantibodies directed against self-antigens. One particular group of these autoantibodies predominantly targets neutrophil proteins and is collectively called anti-neutrophil cytoplasmic autoantibodies (ANCA)^2^ (1–3). Although ANCA can be found in a variety of autoimmune disorders, they are most common in distinct forms of small vessel vasculitis, hence the term ANCA-associated vasculitis (AAV) which includes granulomatosis with polyangiitis (GPA) and microscopic polyangiitis (MPA) (1, 2). In AAV, the presence and antigen-specificity of ANCA can assist in diagnosis and disease stratification (3). For example, ANCA that target proteinase 3 are typically found in GPA patients whereas ANCA against myeloperoxidase (MPO) are predominantly observed in MPA patients (3). However, why autoimmunity develops against these neutrophilic proteins, and whether there are qualitative aspects of the involved proteins that predispose to this, is unknown.

MPO, one of the most common ANCA autoantigens, is an important and abundant enzyme within the host defense system (4). It catalyzes the conversion of hydrogen peroxidase and chloride anions into hypochlorous acid, a toxin with bactericidal properties (5). The protein is abundantly present in the azurophilic granules of neutrophils, and is released into the phagosome to assist in the degradation of opsonized pathogens; ∼30% may be released into the extracellular space upon degranulation (6). Mature MPO exists as a cysteine-linked homodimer, with each monomer comprising a light and heavy chain linked by disulfide bridges, a covalently linked heme group, and five *N*-linked glycosylation sites per heavy chain (7). The glycosylation of MPO has been shown to affect the protein in a number of ways, including the modulation of its enzymatic activity and its interaction with binding partners such as ceruloplasmin (8, 9). Importantly, the glycosylation of MPO has been indicated to affect the interaction with ANCA as well (9, 10).

Because of its importance, the *N*-glycosylation of MPO has been studied previously either at the released glycan level (8) or qualitatively at the glycopeptide level (11). These earlier studies have indicated MPO glycan species to range from high-mannose to complex diantennary (8, 11). Presently, it is not yet known what the relative abundance of the glycoforms is for each of the five MPO sites, and therefore, which species are dominantly expressed. In addition, there is strong evidence that not all relevant glycosylation characteristics of MPO have yet been charted. For instance, a recent glycoproteomics study on pathogen-infected sputum, which also detected three of the MPO glycosylation sites, showed abundant paucimannose species on these sites (12). Similarly, reports indicate that MPO likely carries mannose-6-phosphate residues (M6P) (13, 14). However, these M6P residues were not reported in these recent glycoproteomics efforts, and it remains therefore unclear how abundant the glyco-modifications are and where on the MPO molecule they reside.

**Figure 1.**
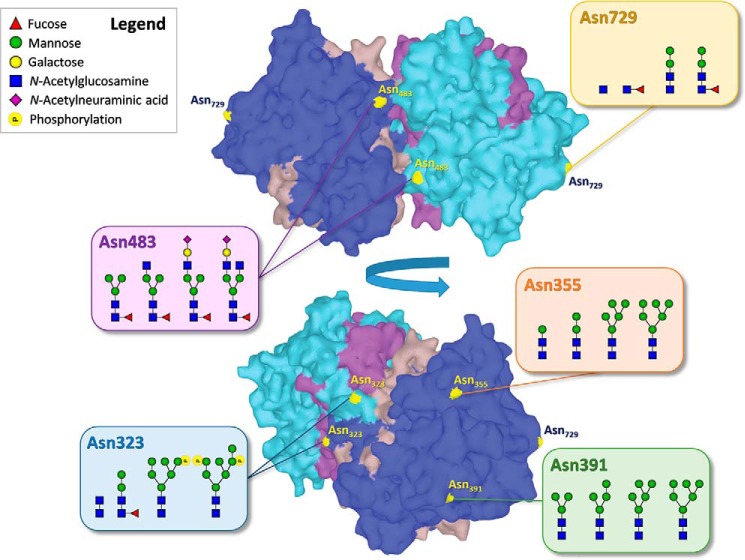
**Summary of the major glycan compositions found on each *N*-glycosylation site of healthy human myeloperoxidase (MPO).** The *light* and *dark blue* regions represent the two heavy chains within the MPO homodimer, the *brown* and *purple* regions the light chains. The structure corresponds to the PDB entry 1CXP (32).

To expand insight into the posttranslational modifications of MPO, we performed a comprehensive glycosylation analysis of MPO derived from neutrophils of healthy human donors, from which we obtained relative glycan occupancies for each independent glycosylation site. To achieve this, we combined contemporary mass spectrometric approaches, namely, high-end bottom-up glycoproteomics and native MS (15, 16). Importantly, recent innovations in MS and bioinformatics have allowed us to better chart M6P residues as well, which so far were often missed (17). In all, we find that on MPO from healthy human donors Asn-355 and Asn-391 mostly harbor high-mannose–type glycans, whereas Asn-483 is the dominant site to display complex-type species. All sites contain a fraction of paucimannose species (and smaller than that), which on Asn-729 are the only species present. Importantly, we demonstrate that M6P is indeed abundantly present on the molecule, predominantly at Asn-323 (Fig. 1).

## Results

**Figure 2.**
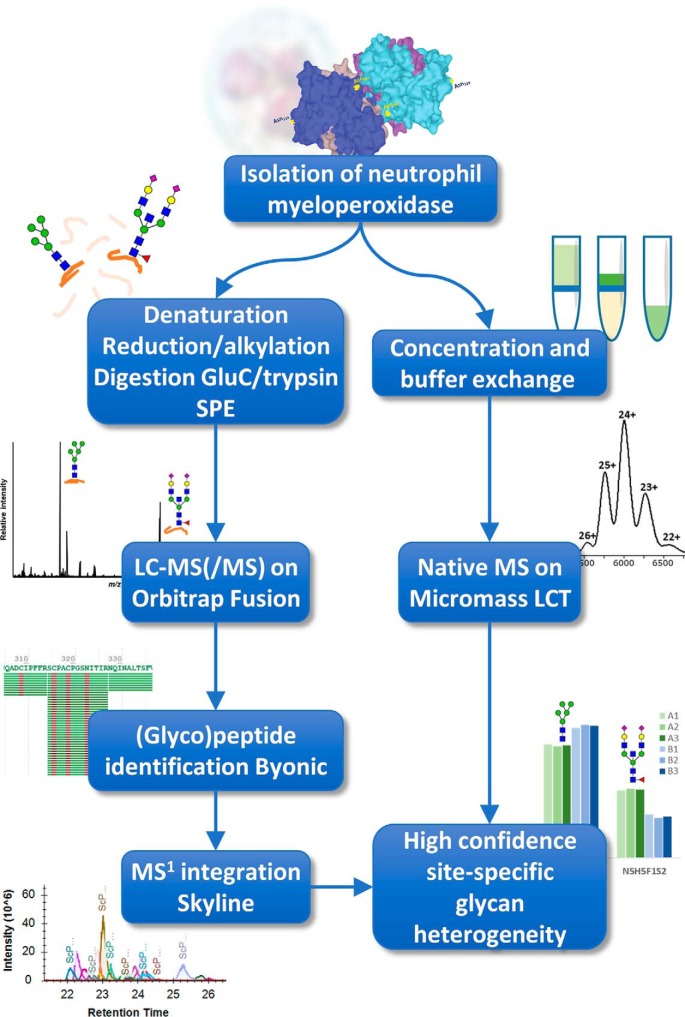
**Experimental workflow.** MPO was isolated from pooled neutrophils of five healthy donors by concanavalin A–Sepharose and size-exclusion chromatography (discovery pool) or only concanavalin A–Sepharose chromatography (validation pool). The resulting material was then analyzed by both a bottom-up (glyco)proteomics approach (*left branch*) and native MS (*right branch*). Confidence in the glycan assignment was achieved by comparing the weighted mass of the bottom-up results with the mass observed by native MS.

We applied native MS and bottom-up glycoproteomics to achieve a comprehensive characterization of MPO glycosylation (Fig. 2). To this end, MPO was purified from healthy donors as described before (18, 19), yielding samples that are typically used in clinical tests for ANCA antigenicity (1). We obtained two pools of MPO, both purified from buffy coats from five healthy individuals, namely 1) a high purity discovery pool for native MS, bottom-up proteomics, and glycan quantification and 2) an independent validation pool of approximate 50% purity to confirm the glycan quantification.

### Bottom-up (glyco)proteomics

Purified MPO was proteolytically digested with both GluC and trypsin and the resulting peptides were analyzed by reversed-phase liquid chromatography (LC)-MS^2^. To improve glycopeptide characterization we applied three fragmentation methods for the analysis of the glycopeptides: 1) higher-energy collisional dissociation (HCD), 2) HCD-product–dependent stepping HCD, and 3) HCD-product–dependent electron-transfer and higher-energy collisional dissociation (EThcD) (16). Triggering of the dependent methods (2 and 3) occurred when glycan-derived fragment ions were detected (Table S1), which included two of the ions that are abundantly generated from M6P-containing glycans (17).

**Figure 3.**
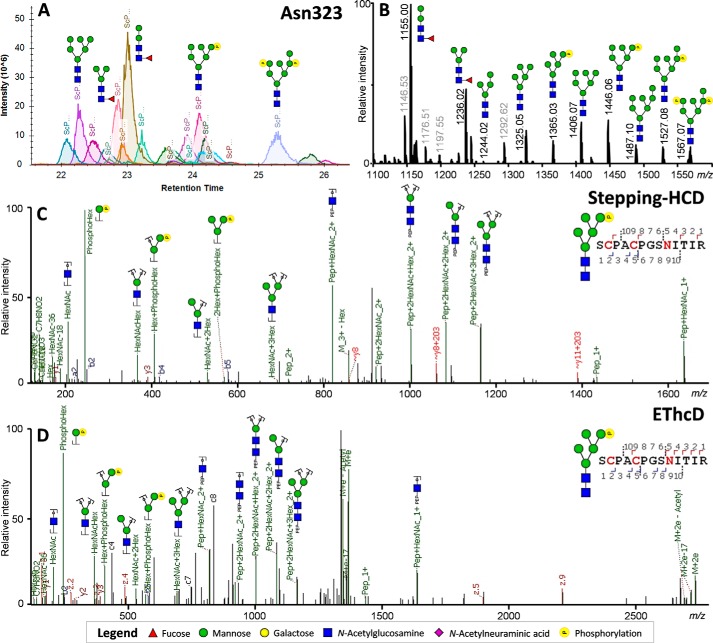
**MS analysis of the MPO glycopeptides covering Asn-323, demonstrating the presence of phosphorylated *N*-glycans.** *A*, chromatographic separation of each of the peptide glycoforms as used for the relative quantification of the peptide glycoforms. *B*, averaged MS^1^ spectra across the retention time window with Asn-323–specific precursor ions (±21–26 min). *Greyed out values* indicate nonglycopeptide signals within the same retention time window. *C*, HCD-product–dependent stepping-HCD fragmentation of the phosphorylated glycopeptide at *m*/*z* 1365.03. *D*, HCD-product–dependent EThcD fragmentation of the phosphorylated glycopeptide at *m*/*z* 1365.03 confirming the assignments.

The spectra were searched with Byonic for the presence of glycopeptides. Multiple sequences of MPO were used for the bottom-up search actions, which included the full sequence obtained from UniProt (P05164), the separate prepeptide, and three main processing variants covering the propeptide, the light chain, and the heavy chain (20). For *N*-glycosylation we allowed 138 glycan compositions that followed the known biosynthetic pathways (Table S2) (20, 21). Inspection of the MS^2^ scans did not reveal evidence of antennary fucosylation (*m*/*z* 350.145 or 512.197 [M+H]^+^), suggesting the absence of Lewis-type structures. Because of this, the glycan compositions were chosen to carry one fucose residue at most. Similarly, no signals were detected that suggested the presence of *N*-glycolylneuraminic acid (*m*/*z* 290.087 or 308.098 [M+H]^+^), therefore, sialylation was presumed to only occur in the *N*-acetylneuraminic acid variant (*m*/*z* 274.092 and 292.103). MS^2^ did show strong indication of phosphohexose residues (*m*/*z* 243.026 and 405.079 [M+H]^+^) (Fig. 3), leading to the inclusion of 34 compositions that adhered to the lysosomal pathway of degradation (*e.g.* HexNAc_2_Hex_5_P_1_ and HexNAc_4_Hex_9_P_2_) (Table S2) (20, 21).

Using Byonic, we achieved a high sequence coverage, namely an MPO light chain coverage of 86.9% (S.D. ± 0.0%; the S.D., calculated from triplicate LC-MS^2^ runs) and a heavy chain coverage of 96.9% (S.D. ± 0.8%). Glycopeptides were matched with a Byonic score cutoff of 150 (22), which allowed for the identification of all potential glycosylation sites and assessment of the relative abundances of the attached glycans (Table S3). Following the curation, no (glyco)peptides were matched that could correspond to the prepeptide or propeptide of MPO, suggesting MPO was exclusively present in its mature form.

**Figure 4.**
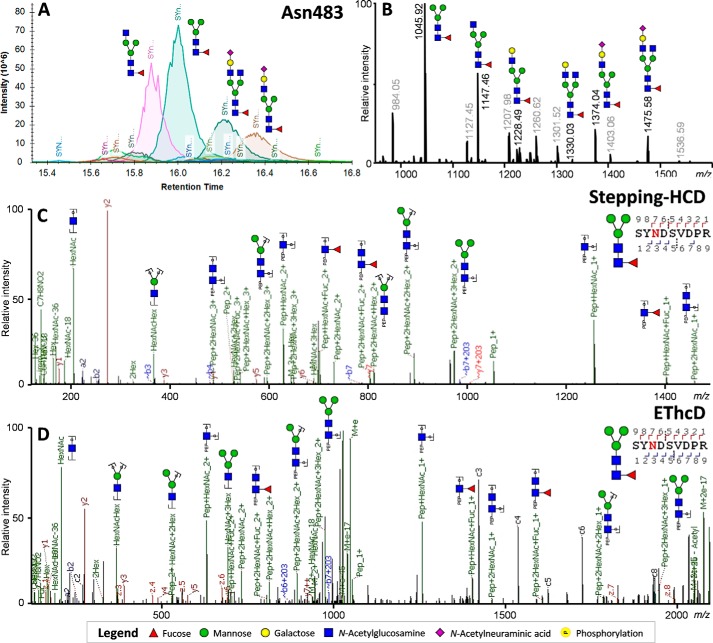
**MS analysis of MPO glycopeptides covering Asn-483, demonstrating the presence of paucimannose-type glycosylation.** *A*, chromatographic separation of each of the peptide glycoforms as used for the relative quantification of the peptide glycoforms. *Greyed out values* indicate nonglycopeptide signals within the same retention time window. *B*, averaged MS^1^ spectra across the retention time window with Asn-483–specific precursor ions (±15–18 min). *C*, HCD-product–dependent of the paucimannose glycopeptide at *m*/*z* 1045.92. *D*, HCD-product–dependent EThcD fragmentation of the phosphorylated glycopeptide at *m*/*z* 1045.92.

The relative glycosylation of each peptide was obtained by use of Skyline, which allowed us to assess the areas within the retention time window of each peptide (Figs. 3*A* and 4*A*). MS^1^ traces were manually inspected and annotated to ensure no complimentary glycan species were excluded from the analysis (Figs. 3*B* and 4*B*). Additionally, MS^2^ scans were manually verified for each main signal to ensure the correct interpretation of the major glycosylation features, which included phosphorylated glycans (Fig. 3, *C* and *D*) and paucimannosidic glycans (Fig. 4, *C* and *D*), as well as to position the fucose on the core-GlcNAc (Fig. 4, *C* and *D*).

The glycopeptide areas resulting from the process of curation and integration were normalized to the sum of intensities for each site. To confirm the biological reproducibility of our observations, we repeated this whole analysis on a second independent batch of partially purified MPO. To inform on the co-purified proteins present in this validation pool, we used Byonic to search the bottom-up proteomics data against a human proteome database. The main co-purified proteins turned out to be lactotransferrin, bactericidal permeability-increasing protein, cathepsin G, neutrophil elastase, and several other proteins that are known to be abundant in neutrophil granules (Table S4). We could find no evidence for the presence of glycosidases or glycosyltransferases in this sample.

### Native MS

MPO was buffer exchanged to 150 mm ammonium acetate (pH 7.5) and analyzed by MS under native conditions. To achieve this, ∼3 μm protein was injected by direct infusion into a Q-Tof TOF-MS system, tuned to provide optimal signal intensity without displaying fragmentation or denaturation.

**Figure 5.**
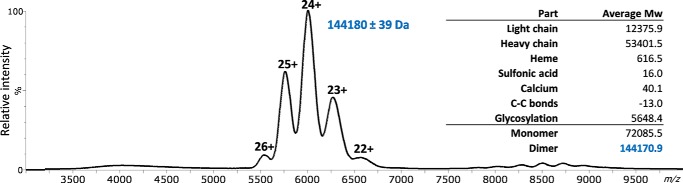
**Native mass spectrum of MPO reveals the exclusive presence of a MPO dimer with an average molecular weight (Mw) of 144,180 Da.** A comparison of this Mw with the theoretical Mw of the dimer, based on the peptide backbone mass and all detected glycosylations, is provided in the *inset*. The average contribution to the mass of the glycosylation was determined from the bottom-up glycoproteomics experiments, overall leading to a high congruency between the theoretical and observed masses.

The highly complex heterogeneity in the MPO glycosylation prevented the acquisition of high-resolution native spectra. Still, we were able to record a charge state–resolved MS spectrum, which allowed us to determine the average molecular weight. MPO was detected as a single ion series with at least five different charge states, ranging from [M+22H]^22+^ to [M+26H]^26+^ (Fig. 5), from which we could obtain an average molecular weight of 144,180 Da (S.D. ± 39 Da).

By adding the theoretical average glycan mass, as calculated from our bottom-up experiments, to the theoretical mass of the MPO peptide backbone, we arrived at a total molecular weight of 144,171 Da for dimeric MPO. This matched, within the error margins, the mass observed by native MS (Fig. 5). The congruence between observed and theoretical molecular weights provided high confidence that MPO was predominantly present in its mature dimeric form (without propeptide and prepeptide segments) and validated our glycan assignment in the bottom-up data. No monomeric and other oligomeric states of MPO were detected.

### Site-specific glycosylation

**Figure 6.**
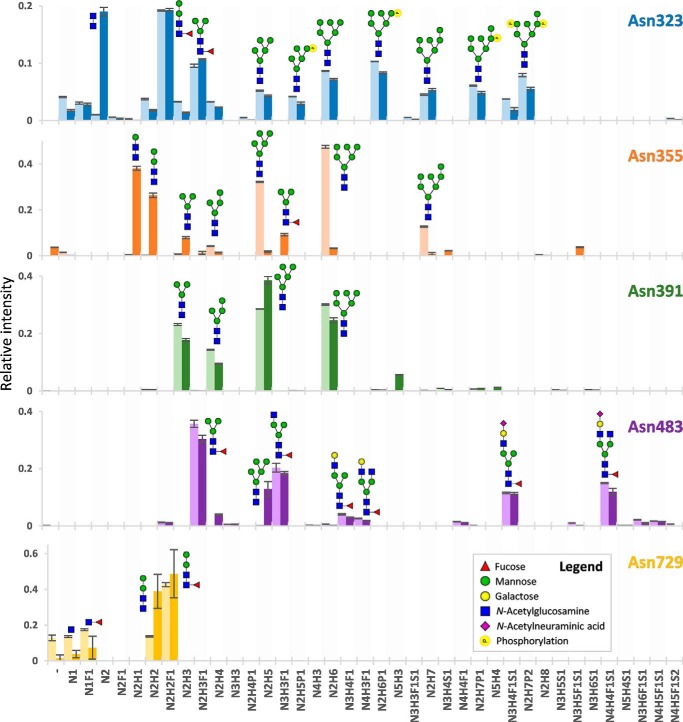
**Qualitative and quantitative overview of the glycan distribution per MPO *N*-glycosylation site.** Each *colored bar* represents one *N*-glycosylation site, the *lighter* and *darker* variant, respectively, the biologically independent discovery pool and replication pool. The height of the *bars* indicates the mean relative area of MS^1^ signals from a triplicate LC-MS^2^ run (the MS^2^ differing in fragmentation scheme) and the *error bars* the S.D. thereof. Informative fragment spectra were acquired for all shown compositions, reporting on the glycan composition, antenna composition, fucose location, and the location of the phosphate. Antenna- and linkage-isomerism were not established by the study, and the here-presented compositions are proposed on basis of the literature on glycan biosynthesis (24, 25) but may also have been formed by glycosidase action. *H* = hexose, *n* = *N*-acetylhexosamine, *F* = deoxyhexose (fucose), *S* = *N*-acetylneuraminic acid, *p* = phosphorylation.

The glycosylation sites of MPO showed remarkably high diversity in occupying glycan species (Fig. 6). Unlike other sites, Asn-323 proved to have 29.0% (S.D. ± 0.4%) occupancy of phosphorylated glycans, in the second pool replicated to be 21.7% (S.D. ± 0.2%). Fragmentation analysis clearly showed the phosphate residues to be associated to hexose residues, apparent from the [M+H]^+^ oxonium ions of Hex_1_P_1_ (*m*/*z* 243.026), Hex_2_P_1_ (*m*/*z* 405.079), and Hex_3_P_1_ (*m*/*z* 567.132) (Fig. 3, *C* and *D*). Together, this strongly suggests the presence of M6P residues on MPO. In contrast, we did not find any evidence for phosphorylation on the peptide at either Ser, Thr, Tyr, or His residues (23). Notably absent within the data were species with additional GlcNAc attached to the phosphate, as would be expected for molecules destined for lysosomal degradation (as observed in other cell types) (17).

The other major glycosylation feature for Asn-323 was the abundant presence of paucimannose glycans (here defined as having fewer hexoses than five and at most two *N*-acetylhexosamines, *e.g.* HexNAc_2_Hex_3_dHex_1_), with a relative abundance of 48.5% (S.D. ± 0.3%) and 59.4% (S.D. ± 0.5%) for the replication pool. Of note was that the paucimannose species were core-fucosylated, unlike the high-mannose glycans, suggesting they may have been formed from truncation of a hybrid or complex *N*-glycan lineage (24, 25).

Both Asn-355 and Asn-391 primarily displayed high-mannose or paucimannose glycans without core-fucosylation. Of interest, the glycan species on Asn-391 proved comparable in size between the discovery and replication MPO (the highest signals belonging to HexNAc_2_Hex_5_ (28.6% S.D. ± 0.0%, replicated as 38.36% S.D. ± 1.3%) and HexNAc_2_Hex_6_ (30.1% S.D. ± 0.2%, replicated as 24.7% S.D. ± 0.8%). The occupancy on Asn-355, on the other hand, differed considerably between the two measured MPO samples, the main species in the discovery MPO being HexNAc_2_Hex_5_ (32.1% S.D. ± 0.2%) and HexNAc_2_Hex_6_ (47.5% S.D. ± 0.6%), whereas in the biological replicate the major signals corresponded to HexNAc_2_Hex_1_ (38.1% S.D. ± 0.9%) and HexNAc_2_Hex_2_ (26.4% S.D. ± 0.9%).

Asn-483 was the only site to show abundant signals of sialylated complex glycans, including HexNAc_3_Hex_4_dHex_1_Neu5Ac_1_ (11.5% S.D. ± 0.3%, and 11.2% S.D. ± 0.4% in replicate) and HexNAc_4_Hex_4_dHex_1_Neu5Ac_1_ (15.0% S.D. ± 0.2% and 12.0% S.D. ± 1.2%) as major species. With this, the total of sialylated species comprised 32.3% (S.D. ± 0.8%, and 26.0% S.D. ± 1.1%). The other dominant species to occupy Asn-483 appeared to be truncated variants of these glycans, namely HexNAc_2_Hex_2_dHex_1_ (35.7% S.D. ± 1.3%, and 30.4% S.D. ± 1.3%) and HexNAc_3_Hex_3_dHex_1_ (20.4% S.D. ± 1.5%, and 18.4% S.D. ± 0.5%). Notably, the vast majority of species on Asn-483 was core-fucosylated, the relative areas hereof summing up to 97.7% (S.D. ± 0.1%, and 81.9% S.D. ± 2.7%).

Finally, core-fucosylation was also high on site Asn-729 (60.1% S.D. ± 1.7% and 56.0% S.D. ± 1.7%), although a high degree of nonfucosylated glycans was also found (27.2% S.D. ± 0.5% and 42.7% S.D. ± 1.7%). The most abundant features of Asn-729 turned out to be HexNAc_2_Hex_2_ (13.7% S.D. ± 0.4%) and HexNAc_2_Hex_2_dHex_1_ (42.6% S.D. ± 1.2%); it also showed the presence of even smaller glycans in the form of HexNAc_1_ (13.5% S.D. ± 0.5% and 3.7% S.D. ± 2.1%) and HexNAc_1_dHex_1_ (17.4% S.D. ± 0.5% and 7.3% S.D. ± 6.4%). Interestingly, among the glycosylation sites, Asn-729 showed the most prominent abundance of the nonmodified peptide (12.8% S.D. ± 1.6%), albeit to a much lesser degree within the biological replicate (1.4% S.D. ± 1.9%).

## Discussion

It remains unknown how autoimmunity develops, and why certain proteins and protein groups are relatively frequently targeted. Factors known to associate with the development of autoimmunity include genetics, HLA type, and instances of infection (26–28), but the quality of the self-antigens invariably plays a role as well (9, 10). As MPO is one of the most commonly targeted neutrophil antigens in AAV, it is of high interest to uncover the nature of its posttranslational modifications.

By the combination of bottom-up glycoproteomics and native MS (29), we here revealed the high abundance of quite peculiar glycosylation features on MPO. Next to demonstrating the complex heterogeneity between the *N*-glycosylation sites, we show the extent of their heavily truncated glycan species, and pinpoint also one particular site which shows abundant phosphomannosylation. Although from a combination of literature the existence of these could already be inferred (8, 11–14), we here for the first time report them concurrently with their relative abundance for each site.

### Uncommon glycosylation

We detected truncated glycans on all glycosylation sites, ranging from paucimannose species down to a single HexNAc with and without fucose. The presence of these uncommon small glycans was heavily scrutinized. We applied three different MS fragmentation strategies to obtain reliable and high-quality MS^2^ data for all observed glycopeptides. By this we could clearly confirm the glycans to be *N*-linked to the Asn residues rather than *O*-linked to Ser or Thr, which would be a possible alternative explanation for the observed smaller glycan compositions. Furthermore, we confirmed the unique retention times for each glycopeptide, making it unlikely that the signals were generated by mass spectrometric decay, and matched the bottom-up assignment to the mass of MPO obtained by native MS, providing confidence in our assignments. Lastly, we supported our data by analyzing a second biological replicate of MPO obtained from a different pool of donors.

Our findings validate previous reports on paucimannose species (11, 12) but expand it to cover all sites of MPO and demonstrate that this truncation process is not stopped at the paucimannose level. We also uncover the presence of a large degree of phosphomannosylated glycans on Asn-323 specifically. The detection of M6P residues has recently been enabled by 1) the improved functionality of the search engine Byonic and 2) our choice to include phosphomannose-specific oxonium ions to trigger MS^2^ (*m*/*z* 243.026 and 405.079) (16, 17).

Although we present here the first mass spectrometric detection of phosphomannose residues on MPO, support for this is available in the literature. As one study shows, the azurophilic granules of neutrophils, the compartments in which MPO typically resides, can be positively stained for the presence of M6P residues (13). Secondly, capillary zone electrophoresis of glycans released from MPO has shown a fraction of the peaks (6.2%) to disappear after alkaline phosphatase treatment, requiring at least some M6P to be present (14). Combining this earlier evidence with ours, it becomes apparent that M6P residues are present on MPO *in vivo*. Strikingly, although the total amount of phosphorylation may be relatively low (6.2% of glycan species, as reported previously) (14), our study shows that these groups are primarily located at a single site within MPO, namely Asn-323. This means that the M6P abundance of that single site, namely 30% of the glycan area, translates to M6P being present on 30% of MPO monomers.

Phosphomannosylation is a process which typically occurs in the Golgi apparatus to direct glycoproteins toward the lysosomal pathway of degradation (25). These lysosomes then perform proteolysis and harbor also enzymes that can degrade glycans to the truncated states we observed in our study. Neutrophils, however, do not have classical lysosomes (13), and it must be noted that the native mass we observed corresponded to a fully formed dimeric MPO without apparent peptide degradation. We hypothesize therefore that neutrophils may have repurposed the M6P-mediated trafficking from the lysosomal pathway to populate the contents of its azurophilic granules. If true, this would mean that most proteins within the azurophilic granules, MPO and otherwise, would contain at least one site with abundant phosphomannosylation. However, verification of this would require further study.

In line, our detection of paucimannose (and smaller) glycosylation may be a consequence of glycosidases present in the azurophilic granules, which include α- and β-mannosidases (12, 30). As such, the glycosylation we observe on MPO may be the result of progressive exposure to glycosidase enzymes present in the granule environment, or in the extracellular environment following degranulation. Although neutrophils are typically short-lived cells, it may be that the older population expels MPO into the extracellular environment that carries smaller glycan species than otherwise, or that MPO glycans are rapidly degraded by the surrounding enzymes.

### Site-dependent heterogeneity

Another intriguing observation from our data is that each of the MPO *N*-glycosylation sites exhibits a different dominant range of glycans, even though they have followed the same path through the endoplasmic reticulum, Golgi apparatus, and granule maturation (Fig. 6). Phosphomannose species are restricted to Asn-323, complex/hybrid species are present on Asn-483 and Asn-729, and species of various mannose-size are located on Asn-355 and Asn-391. Each site has the capacity to carry truncated species as well and does so in varying degrees. This heterogeneity of glycosylation cannot exist without interplay between the (de)glycosylating environment and MPO.

**Figure 7.**
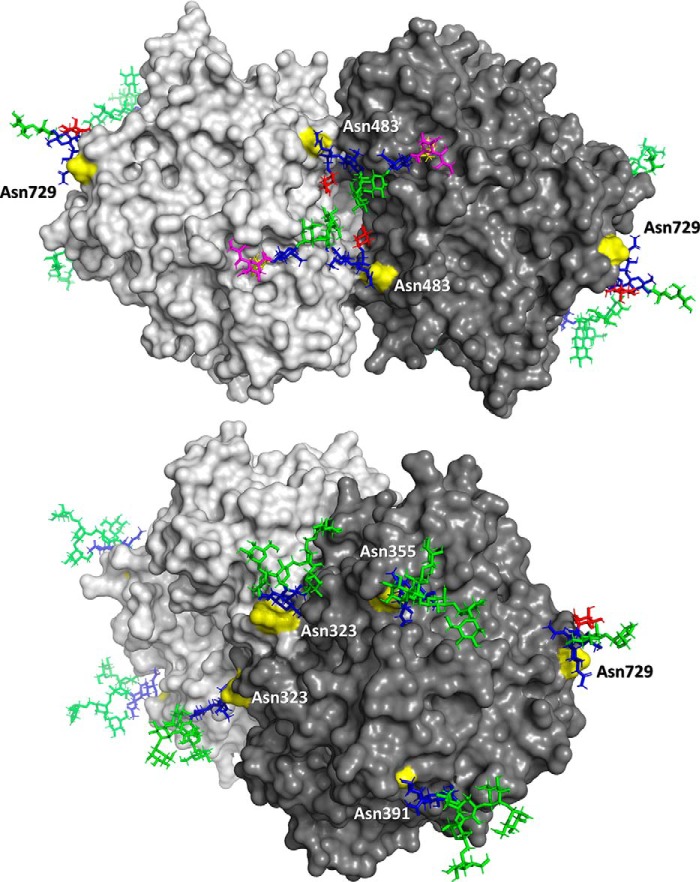
**Overview of representative glycan structures as modeled on the backbone of the MPO dimer.** Linkages of the glycan species were not known from the study, but were estimated on basis of the literature (24, 25). Note that glycan structures should not be regarded as static, but are highly dynamic and will occupy a broad area surrounding the presented models.

Both Asn-323 and Asn-483 are on the interface between the MPO monomers and have been implicated in stabilizing the dimer by interaction with the opposing molecule (Fig. 7) (31–33). Sufficient interaction of the glycan with the monomer on the other side would provide some protection of the glycan against glycosidase action. This might, for example, explain why the sialylated glycans at Asn-483 appear rather asymmetric, one antenna might interact with (and be protected by) the peptide backbone whereas the other one could be free and accessible to glycolysis.

It is similarly remarkable that Asn-355 and Asn-391 carry only paucimannose and high-mannose–type glycosylation (31–33) and that they have not been modified into complex or hybrid species. This must mean that they were not accessible to processing in the Golgi apparatus, and that they have likely been protected either by their own interaction with the MPO backbone or by a chaperone protein. Between these, Asn-355 was the site to differ most between the first and second biological replicate, with the base species, respectively, being HexNAc_2_Hex_6_ or HexNAc_2_Hex_1_. An explanation for this difference may be that one of the samples contained a dominant donor with a particular deviating glycosylation phenotype or, alternatively, that the second replicate received more exposure to glycosidase enzymes in the neutrophil granule or during sample preparation. A proteomics analysis on the second replicate revealed no evidence for the presence of glycosidases, however, and none of the other glycosylation sites showed a similar discrepancy in glycan occupancy.

### Immunological implications

The uncommon glycosylations we observe on MPO also lead to several hypotheses on how the binding between MPO and MPO-ANCA might be affected. For one, ANCA might directly interact with the uncommon glycan epitopes (or in combination with the local protein backbone structure). A recent study supports this hypothesis, as it describes loss of antigenicity after enzymatic deglycosylation of MPO (9). The inverse situation may also be true, with glycans normally covering and protecting sites from recognition. Small glycans may not be large enough to obscure particularly vulnerable sites, thereby predisposing to ANCA generation (Fig. S1). A recent study demonstrated a number of antigenic sites to be only recognized with sufficiently small *N*-glycan species (GlcNAc_2_ and smaller), and not with larger ones (10). Within this context, it is important to see that the very small glycan compositions that lead to the antigenicity appear to occur on human MPO *in vivo*.

Alternatively, an effect of glycosylation may lie in modulating the 3D structure of the MPO dimer, or its stability and flexibility (*e.g.* by the species on interface sites Asn-323 and Asn-483). A flexible protein can more easily present uncommon (structural) epitopes than a stable one, which may play a particular role in the development and recognition of conformational epitopes (34).

Lastly, many cells carry an array of lectins and M6P-receptors for internalizing proteins that respectively exhibit truncated or M6P-carrying glycans (14). Following our observations, this may be to facilitate internalization and removal of MPO, and possibly other proteins in the toxic neutrophil granule mixture.

In all, it is likely that the glycosylation of MPO plays a role in its recognition by ANCA, yet the same glycan may have a protective or immunogenic effect depending on the circumstance. As such, we propose the inclusion of thorough glycan characterization in future studies on protein-specific ANCA antigenicity.

## Conclusion

To conclude, we have performed, using biological replicates, an in-depth glycosylation analysis of human MPO, and report the relative abundance of all glycoforms on each of the glycosylation sites. Observed atypical aspects of glycosylation include highly truncated paucimannose species and smaller, abundant phosphomannosylation on one particular site, and a highly complex heterogeneity in between sites. We believe this report aids in our understanding of how MPO might be regulated, and we advocate further study into how this may contribute to its antigenicity.

## Experimental procedures

### Chemicals and reagents

Tris-HCl, tris(2-carboxyethyl)phosphine (TCEP), chloroacetamide, sodium deoxycholate (SDC), TFA, and trypsin were purchased from Sigma-Aldrich. GluC was obtained from Roche. Acetonitrile (ACN) was acquired from Biosolve (Valkenswaard, The Netherlands). Finally, water was generated from a Q-POD or Q-Gard 1 system (Millipore), operated at ≥ 18.2 megaohms.

### Purification of myeloperoxidase

Buffy coats were obtained as anonymized coded specimens from the Dutch blood bank (Sanquin). MPO was purified from two separate pooled buffy coats of five donors each (respectively, the discovery and replication pool) following established protocols (18, 19). Briefly, polymorphonuclear (PMN) leukocytes were isolated from the buffy coats on a Ficoll density gradient followed by dextran sedimentation (18, 19). A PMN lyosomal extract was prepared by dissolving PMNs in cetyltrimethyl ammonium bromide (Sigma) and sonification. To capture the MPO fraction, the discovery pool was further purified by concanavalin A-Sepharose and Sephadex G100 size-exclusion chromatography, whereas the replication pool was only purified by concanavalin A-Sepharose chromatography. The final MPO preparation of the discovery pool had an OD 428/280 ratio of 0.76 and a total MPO concentration of 0.434 mg/ml as calculated using the extinction coefficient for MPO, ϵ_428_ = 91,000 M^−1^ cm^−1^ per heme. The replication pool had an OD 428/280 ratio of 0.5 and a total MPO concentration of 0.398 mg/ml.

### Sample preparation for native MS

Native MS was performed as described previously (35). Specifically, the enriched MPO (discovery pool) was buffer exchanged to 150 mm ammonium acetate, pH 7.5, using a Vivaspin 500 30 kDa molecular weight cutoff filter (Sartorius Stedim Biotech, Goettingen, Germany) by 10 × 15 min centrifugation at 15,000 × *g*. No significant sample loss was detected during the centrifugation as the green-colored MPO was retained by the filter and was not visible in the flow-through. Following buffer exchange, the MPO was adjusted to an approximate concentration of 3 μm with 150 mm ammonium acetate, pH 7.5.

### Native MS

Native MS was performed on a Micromass LCT mass spectrometer (Waters), calibrated using a 25 mg/ml CsI solution. Electrospray ionization was achieved from a gold-coated glass capillary, employing a capillary voltage of 1.2 kV, a sample cone voltage of 100 V, an extraction cone voltage of 10 V, a source temperature of 80 °C, and a source pressure of 8.8 mbar. Spectra were summed across 5 min, and smoothed for visualization purposes, whereas the *m*/*z* values were obtained from the centroids at 70% intensity.

### Sample preparation for LC-MS^2^ (glyco)proteomics

MPO (discovery and replication pool**)** was denatured, reduced, alkylated, and proteolytically digested with GluC and trypsin, as described previously, but with minor deviations (36). Briefly, 10 μg of MPO was brought to 100 mm Tris-HCl, pH 8.5, 5 mm TCEP, 30 mm chloroacetamide, and 1% SDC. This mixture was incubated for 4 h at 37 °C with GluC with an enzyme:protein ratio of 1:75 w/w, followed by an overnight incubation at 37 °C with trypsin (1:100 w/w). Hereafter, the SDC was precipitated by bringing the samples to 0.5% TFA and centrifugation at maximum speed for 10 min. The supernatant was collected for subsequent solid-phase extraction.

For solid-phase extraction, we made use of an Oasis μElution HLB 96-well plate (Waters, Wexford, Ireland) positioned on a vacuum manifold. The plate was conditioned with ACN, equilibrated with 0.5% TFA, loaded with the supernatant, and washed with 0.5% TFA, and peptides were finally eluted with 50% ACN 0.5% TFA. The recovered eluate was dried by means of rotary evaporation and reconstituted in 2% formic acid for subsequent LC-MS^2^ analysis.

### LC-MS^2^ analysis

For each of the digested and desalted samples, 100 ng was analyzed by use of an Agilent 1290 Infinity HPLC system (Agilent Technologies, Santa Clara, CA), equipped with flow-splitter to achieve nanoflow, hyphenated to an Orbitrap Fusion Tribrid Mass Spectrometer (Thermo Fisher Scientific). The samples were separated on a 2-cm trap column (100 μm inner diameter, packed with 3 μm ReproSil-Pur C18-AQ; Dr. Maisch GmbH, Ammerbuch-Entringen, Germany) coupled to a 50-cm analytical column (50 μm inner diameter, packed with 2.7 μm Poroshell 120 EC-C18; Agilent Technologies, Santa Clara, CA). For an overview of the buffers, flow rates, and gradient program see Table S5. Mass spectrometry was performed in positive ion mode, with electrospray ionization from a coated fused silica emitter at 2 kV spray voltage.

Each sample was measured in triplicate, using equal MS^1^ acquisition methods, but different MS*^2^* methods. For the MS^1^ scans, the mass range was set from *m*/*z* 350 to 2000 with a resolution of 60,000, an AGC target of 400,000 with maximum injection time of 50 ms. Each of the three MS^2^ methods initiated HCD fragmentation (30% normalized collision energy) on the highest charge state, lowest *m*/*z* signals within a 3-s cycle time, using an exclusion time of 30 s. MS^2^ by HCD was recorded with a resolution of 30,000 from *m*/*z* 120 to 4000, with an AGC target of 50,000 and a maximum injection time of 50 ms. For MS^2^ method 1, only HCD fragmentation was performed. For MS^2^ method 2, the detection of at least three oxonium ions within the HCD spectrum triggered stepping-HCD on the same precursor signal, combining the HCD fragments with normalized collision energy of 10, 25, and 40%. For the full list of triggering oxonium ions see Table S2). Stepping-HCD was recorded with a resolution of 30,000 from *m*/*z* 120 to 4000, with an AGC target of 200,000 and a maximum injection time of 250 ms. For MS^2^ method 3, detection of the oxonium ions triggered EThcD (30% supplemental activation), which was recorded with a resolution of 30,000 from *m*/*z* 120 to 4000, with an AGC target of 200,000 and a maximum injection time of 250 ms.

### Data analysis

For data analysis we made use of the UniProt sequence of human MPO (P05164), as well as the peptide sequences that result from intracellular processing of the protein (20, 21) (for the full list of FASTA sequences see Table S6). To search the validation pool against the human proteome we made use of the Swiss-Prot database, filtered for human entries.

Bottom-up data were interpreted with Byonic v3.3.11 (Protein Metrics Inc.), updated to include assignment of phosphomannose oxonium ions (17, 37). Raw data were searched with C-terminal cleavage sites at Arg and Lys (trypsin), and Glu and Asp (GluC). We allowed for three missed cleavages, using a precursor mass tolerance of 10 ppm and fragment mass tolerance of 20 ppm. Cys carbamidomethylation was included as fixed modification, and Met oxidation as variable modification. No *O*-glycosylation or other posttranslational modifications were detected in the bottom-up data and were consequentially not included in the final search actions. For *N*-glycosylation we included 138 compositions following the pathways for *N*-glycan biosynthesis (Table S2) (24, 25). To limit the search space, only one deoxyhexose was allowed per glycan, which, by fragmentation, was confirmed to be exclusively positioned on the core *N*-acetylhexosamine.

Skyline (v3.7.0.11317) was used to perform relative quantification (38). For this, we integrated each of the peptides found glycosylated by Byonic (Table S7), which included all major miscleavages and oxidation variants (for the list of included peptides see Table S6). For each of these, the aforementioned 138 glycan compositions were integrated from each LC-MS^2^ run (Table S8). Integrations obtained as such were subsequently curated to adhere to the following criteria: 1) ≤ 5 ppm error to the theoretical mass, 2) having an idotp of ≥ 0.85 with the theoretical isotopic pattern, 3) eluting within ±2 min of the mean retention time for that peptide, and 4) having no apparent overlapping isotopic patterns. The resulting list of glycopeptides was in agreement with the annotation by Byonic, and further used for relative quantification. For each of the curated peptide glycoforms, the MS^1^ areas were integrated and peptides informing on the same *N*-glycosylation site combined. The resulting areas were normalized to the sum for each *N*-glycosylation site (Table S9). Bar graphs were constructed from the relative intensities arising from the MS^1^ triplicate, with error bars showing the S.D. thereof.

For comparison with native MS, the average mass of each glycopeptide, calculated from its chemical composition, was multiplied by its relative abundance. The total mass of glycosylation originates from adding the average glycan masses of each glycosylation site, doubled to account for the dimerization of MPO.

### Data visualization

For visualization of glycan species we followed the recommendations of the Consortium for Functional Glycomics (39). Glycan cartoons were constructed using GlycoWorkbench (v2.1 build 146) (40).

PDB entry 1CXP was used for visualization of the MPO structure (32). To model the glycans on top hereof we made use of the Glycam prediction tool (www.glycam.org),^3^ whereby the given glycans were energy-minimized using AMBER (41, 42). GlcNAc_2_Man_7_ was added to both instances of sites Asn-323, Asn-355 and Asn-391, and Fuc_1_GlcNAc_3_Man_3_Gal_1_Neu5Ac_1_ was added to Asn-483 and Fuc_1_GlcNAc_2_Man_3_ to Asn-729. For the high-mannose species we assumed an equal mannose occupancy across branches. The site to contain complex-type glycosylation, Asn-483, was specifically modeled with a β1,2-linked antenna on the α1,6-arm, which minimized to a groove on the opposing monomer, whereas other plausible linkages led to steric clashes.

All generated models were visualized with PyMOL (43). Glycan residues were colored according to the CFG recommendations. The surface of the MPO dimer was either colored by monomer and chain, or by the previously reported conformational epitopes recognized by MPO-ANCA in AAV patients (Fig. S1) (34).

### Data availability

All raw data and processed files have been made available through the MassIVE repository and assigned the identifier MSV000084281.

## Author contributions

K. R. R., P. H., and A. J. R. H. conceptualization; K. R. R. data curation; K. R. R. formal analysis; K. R. R. and V. F. investigation; K. R. R. visualization; K. R. R. writing-original draft; K. R. R., V. F., M. G. H., E. B., P. H., and A. J. R. H. writing-review and editing; V. F., E. B., P. H., and A. J. R. H. supervision; M. G. H., E. B., P. H., and A. J. R. H. resources; M. G. H., E. B., P. H., and A. J. R. H. methodology; E. B., P. H., and A. J. R. H. funding acquisition; A. J. R. H. software; A. J. R. H. project administration.

## Supplementary Material

Supporting Information

## Footnotes

3 Please note that the JBC is not responsible for the long-term archiving and maintenance of this site or any other third party hosted site.

## Footnotes

2 The abbreviations used are:

ANCA: anti-neutrophil cytoplasmic autoantibodies
AAV: ANCA-associated vasculitis
MPO: myeloperoxidase
M6P: mannose-6-phosphate
HCD: higher-energy collisional dissociation
EThcD: electron-transfer and higher-energy collisional dissociation
TCEP: tris(2-carboxyethyl)phosphine
SDC: sodium deoxycholate
ACN: acetonitrile
PMN: polymorphonuclear.
