# Supplementary material for: Neutrophil myeloperoxidase harbors distinct site-specific peculiarities in its glycosylation
Source: J Biol Chem. 2019 Nov 12;294(52):20233–45. doi: 10.1074/jbc.RA119.011098 (PMC6937560; doi:10.1074/jbc.RA119.011098)
Supplement: Supporting Information [file supp_294_52_20233__index.html]

Neutrophil myeloperoxidase harbors distinct site-specific peculiarities in its glycosylation — The glycosylation of neutrophil myeloperoxidase — Neutrophil myeloperoxidase harbors distinct site-specific peculiarities in its glycosylation — The glycosylation of neutrophil myeloperoxidase — Supporting Information 

# Neutrophil myeloperoxidase harbors distinct site-specific peculiarities in its glycosylation

## Supporting Information

- Supplemental Figure 1 - Supplemental Figure 1
- Supplemental Tables 1-8 - Supplemental Tables 1-8
