# Supplementary material for: Neutrophil myeloperoxidase harbors distinct site-specific peculiarities in its glycosylation
Source: J Biol Chem. 2019 Nov 12;294(52):20233–45. doi: 10.1074/jbc.RA119.011098 (PMC6937560; doi:10.1074/jbc.RA119.011098)
Supplement: Supporting Information [file supp_RA119.011098_155883_1_supp_424106_q0h11k.pptx]

## Slide 1
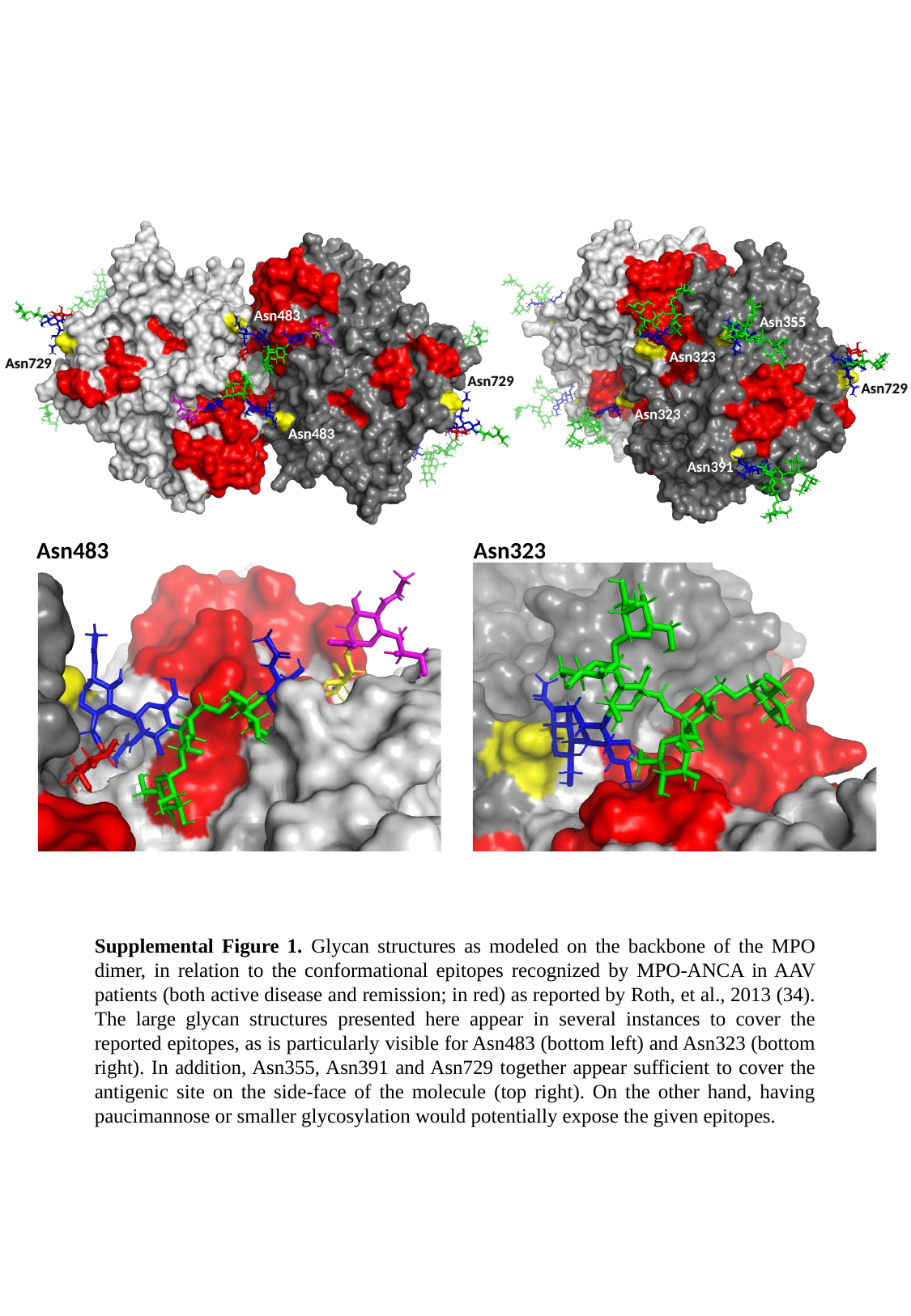

Asn483
Asn355
Asn323
Asn729
Asn729
Asn729
Asn323
Asn483
Asn391
Asn483
Asn323
Supplemental Figure 1. Glycan structures as modeled on the backbone of the MPO dimer, in relation to the conformational epitopes recognized by MPO-ANCA in AAV patients (both active disease and remission; in red) as reported by Roth, et al., 2013 (34). The large glycan structures presented here appear in several instances to cover the reported epitopes, as is particularly visible for Asn483 (bottom left) and Asn323 (bottom right). In addition, Asn355, Asn391 and Asn729 together appear sufficient to cover the antigenic site on the side-face of the molecule (top right). On the other hand, having paucimannose or smaller glycosylation would potentially expose the given epitopes.
